# Levy Equilibrium Optimizer algorithm for the DNA storage code set

**DOI:** 10.1371/journal.pone.0277139

**Published:** 2022-11-17

**Authors:** Jianxia Zhang

**Affiliations:** School of Intelligent Engineering, Henan Institute of Technology, Xinxiang, China; Sichuan University, CHINA

## Abstract

The generation of massive data puts forward higher requirements for storage technology. DNA storage is a new storage technology which uses biological macromolecule DNA as information carrier. Compared with traditional silicon-based storage, DNA storage has the advantages of large capacity, high density, low energy consumption and high durability. DNA coding is to store data information with as few base sequences as possible without errors. Coding is a key technology in DNA storage, and its results directly affect the performance of storage and the integrity of data reading and writing. In this paper, a Levy Equilibrium Optimizer (LEO) algorithm is proposed to construct a DNA storage code set that satisfies combinatorial constraints. The performance of the proposed algorithm is tested on 13 benchmark functions, and 4 new global optima are obtained. Under the same constraints, the DNA storage code set is constructed. Compared with previous work, the lower bound of DNA storage code set is improved by 4–13%.

## 1. Introduction

With the rapid progress of science and technology and the increasing popularity of high-speed network, network data, mobile data, social data and other digital information data are increasing exponentially. According to IDC, the total amount of global data will reach 175ZB by 2025. Storage devices based on physical media can not cope with the explosive growth of data. Therefore, how to store these massive data has become a key problem for the long-term sustainable development of information technology. As a new storage method, DNA data storage technology plays an important role in saving storage energy and promoting the development of big data storage. The idea of using DNA molecules to store and store information appeared as early as the 1960s. Since it was difficult to read and write DNA information, it was not until 1988 that Davis [1] began to use DNA to store a small amount of information, but the storage information was very small. In recent years, with the rapid reduction of the cost of base synthesis and the development of DNA sequencing technology, some more practical work has appeared. In 2012, Church et al. [2] adopted A binary model to encode and store digital information by using bases A and C to represent 0 in binary and bases G and T to represent 1 in binary. In order to reduce the error rate, it is required to avoid 4 or more consecutive same bases in the coding information, and ensure the stability of GC content. The compiled DNA sequence is synthesized into several short DNA fragments. Then, through second-generation sequencing, the content of the synthesized DNA fragment is read out, and finally converted into a short fragment, and the position of the fragment in the whole file is found according to the bar code to obtain the original file. Goldman et al. [3] adopted ternary coding model based on Church’s work, that is, each bit of information is represented by 0, 1 and 2 states. They used homopolymer-free DNA sequences to encode ternary digital information. The scheme proposed by Goldman has certain error correction ability, so it can effectively reduce the error rate compared with Church’s work when reading the information stored in DNA. Yazdi et al. [4] described the first DNA-based storage architecture that allowed random access to blocks of data and the rewriting of information stored anywhere within the blocks. The system is based on new constraint coding techniques and corresponding DNA editing methods to ensure data reliability, specificity and access sensitivity. Ceze et al. [5] proposed an architecture for a DNA-based archival storage system. The team managed to encode data from four image files into the nucleotide sequences of synthetic DNA fragments, and they were also able to retrieve the correct nucleotide sequences from a larger pool of DNA and reconstruct the image without losing a single byte of information. Shortly afterward, Microsoft announced that it had saved about 200 megabytes of data using DNA storage technology, including "War and Peace" and 99 classic literary works. In 2017, Erlich and Zielinski [6] proposed the Coding method of DNA fountain algorithm to achieve error-free storage and achieve the coding rate of 1.6bit/nt. In the same year, Shipman et al. [7] introduced images and videos encoded as DNA sequences into the genome of EScherichia coli and read corresponding images and videos from the genome of living bacterial cells. In 2018, the Grass team [8] encoded and stored 35 different files in over 13 million DNA oligonucleotides and could recover each file lossless using random methods. A large primer library has been designed and validated, which can independently recover all files stored in DNA. DNA as a long-term storage medium has been preliminarily demonstrated for storage potential [9–12].

DNA coding is a key technology in DNA storage, which aims to store data information with as few base sequences as possible without error. The results of DNA coding directly affect the performance of storage and the integrity of data read and write. Reasonable and efficient coding is very important for the whole DNA storage system. In 2012, Church et al. [2] used DNA synthesis technology and second-generation sequencing technology to encode 0.65MB of abiotic information into DNA sequence, which was the first application of binary model and achieved an information storage density of 0.83 bit/nt. With the in-depth research on DNA coding, Ross et al. [13] reported that replacement and deletion errors would increase significantly when the running length of homomer exceeded 6. On the other hand, DNA chains with too high or too low GC content were more prone to synthesis and sequencing errors. Due to the above reasons, Bornholt et al. [14] adopted XOR coding principle to improve Church’s coding scheme, which not only realized random access but also achieved 0.85 bit/nt storage density. In terms of error correction coding, Blawat et al. [15] introduced forward error correction to achieve a storage density of 1.08bit/nt. Not long after, Yazdi et al. [4] overcame the need for full sequencing when reading data, designed a coding method to achieve random access through address bit addressing, and a platform for efficient sequencing through iterative alignment and deletion of error check codes, thus achieving high storage density. In 2017, Erlich et al. [6] creatively designed a "fountain code" for information storage, which is highly robust and efficient because it can avoid GC content with high deviation and the generation of homomeric. This is the first time that fountain code introduced in communication coding makes net information density as close to Shannon limit as possible, and shows that error detection/correction algorithm is not necessary for error correction, and the same effect can be achieved by screening sequences. Jeong et al. [16] further improved the fountain code and obtained better decoding results by clustering. Wang et al. [17] designed an encoding method consisting of repeated additive codes (RA) and an efficient hybrid mapping scheme to achieve a storage density of 1.67bit/nt. Zhang et al. used combinatorial constraints to screen DNA storage codes [18–20], and used heuristic algorithms such as CLGBO [21] and NOL-HHO [20] to construct DNA storage code sets, constructing DNA storage code sets of higher quality. Yehezkeli et al. [22] consider noise introduced entirely by uniformly repeated sequences and exploit the relation with equal weight integers in the Manhattan metric. The existence of full-rate reconstruction codes is proved using hyperplane restricted multifaceted wall intersections [23], and a method for the construction of a class of reconstruction codes is given. Lenz et al. present a storage model for disordered sequence representations, deriving a Gilbert-Varshamov lower bound on the reachable bases of error-correcting codes and an upper bound on spherical wrappers [24]. In 2021, Zan et al. [25] proposed a hierarchical error-correction strategy for text DNA storage based on divide-and-conquer algorithm to complete lossless storage of text.

Heuristic algorithms can provide a feasible solution for each instance of the combinatorial optimization problem to be solved at an acceptable cost. The deviation degree of the feasible solution from the optimal solution cannot be predicted in general. Classical heuristic algorithms include genetic algorithm [26], particle swarm optimization [27] algorithm, etc. New ones include the Monarch Butterfly Optimization (MBO) [28], Slime Mould Algorithm (SMA) [29], Moth Search Algorithm (MSA) [30], hunger games search (HGS) [31], RUNge Kutta method (RUN) [32], colony predation algorithm (CPA) [33], Weighted mean of vectors (INFO) [34] and Harris Hawks Optimization (HHO) [20]. They are widely used in engineering to optimize traditional complex engineering problems such as distribution system [35], power flow [36], and power grid [37]. It is also often applied to solve optimization problems in the biological field [38, 39].

The DNA storage coding problem can be equivalent to the DNA coding screening problem satisfying the combinatorial constraints. However, because of the high complexity of the computational process of constraints, the efficiency of using traditional algorithms is too low. Due to the problems of low base utilization and low coding quality in existing DNA storage coding methods, this work constructed a DNA storage coding set that met the combination constraints through the improved LEO (Levy Equilibrium Optimization) to ensure both coding efficiency and coding quality. The LEO algorithm improved by Levy optimizer reduces the possibility of the original algorithm falling into local optimum, and improves the convergence speed of the algorithm. It is possible to construct more sets of DNA storage codes that satisfy the constraints. The constructed encoding set satisfies the Hamming distance constraint, the GC content constraint and the No-runlength constraint, and has some error correction capability. It also offers many coding advantages such as high robustness, low coding complexity and shorter coding time.

## 2. Encoding constraints

### 2.1 Hamming distance constraint

The Hamming distance can be used in other research areas, such as in coding theory, to measure the similarity of two codewords. In DNA storage, a smaller Hamming distance in coding [40] can indicate that there are many identical bases between two different DNA codewords, i.e., an increased possibility of non-specific hybridization. For two different DNA codes *j*, *k*, *HD(j*, *k)* denotes the number of different bases at position *i* of sequence *j*, *k*. The Hamming distance constraint expression can usually be expressed by the following mathematical formula with *HD(j*, *k)* ≥ *d*. The Hamming distance is calculated as follows:

$$HD(a,b)=\sum_{i=1}^{n}hd(a_{i},b_{i}),hd(a_{i},b_{i})=\left\{\begin{array}{l} 0,a_{i}=b_{i}\\ 1,a_{i}\neq b_{i} \end{array}\right.$$ (1)

### 2.2 GC content constraint

A, T, C and G are the four bases that constitute the structure of DNA, among which A and T can form A double-stranded structure when they are complementary, as can G and C. In actual biological operations, sequences with extreme GC content are unstable, so sequences are generally designed according to 40%-60% GC content, which is the GC content constraint condition [41].


$$GC(x)=\frac{\left|G|+|C\right|}{\left|x\right|}$$ (2)

### 2.3 No-runlength constraint

Continuous bases lead to the instability of the molecular structure of the whole sequence, and the hybridization reaction is difficult to control. Errors are especially prone when reading long homopolymers. Therefore, in the coding process, we use No-runlength constraints [42] to try to avoid similar errors. Running the same nucleotides over long periods of time can cause errors in the DNA code. For example, TCCCCAC, C is repetitive, so it is easy to read long C into short C in synthesis and sequencing, resulting in an increase in the error rate of DNA storage information and a decrease in read and write coverage. For code words *L* (*l*_*1*_, *l*_*2*_, *l*_*3*_… *l*_*n*_) is the length of *n*, and for any *I*:

$$\begin{array}{ll} {\text{L}}_{\;\text{i}}\neq {\text{L}}_{\text{i+1}} & \text{i}\in [1,\text{n-1}] \end{array}$$ (3)

## 3. Algorithm description

### 3.1 Equilibrium optimizer

Equilibrium optimization algorithms are inspired by a variety of phenomena in physics, such as mixed dynamic mass balances. The mass balance equation in the mixed dynamic mass balance weight is used to describe the dynamic equilibrium process that limits the concentration of non-reactive substances in the volume. The mass balance equation has the role of providing a fundamental physical explanation for the conservation of mass entering, leaving and arising in the control volume. More detailed information related to the mixed dynamic mass balance process can be found further in the original paper [43].

The steps of EO algorithm are as follows:

Step 1: Initialization
  Initialization is performed according to the multiple parameters in the search space, and the initial concentration is constructed using the number and dimension of uniformly random initialized particles with the following mathematical equation:
$${\vec{v}}_{i}=e_{\text{min}}+\left(e_{\text{max}}-e_{\text{min}}\right)*r_{1}\;i=0,1,2,\ldots ,n$$ (4)
  Here ${\vec{v}}_{i}$ represents the concentration vector of particle *i*, *c*_max_, *c*_min_ representing the upper and lower bounds of the dimension respectively. *r*_*1*_ represents a random vector between [0,1] and contains *n* groups of particles.
Step 2: Balance pool and candidate pool
  Population intelligence algorithms such as the EO algorithm and the particle swarm ant colony algorithm are population-based algorithms. These algorithms divide the search process into two phases: exploration and exploitation. Each algorithm has a different approach to exploration and exploitation. For all heuristic algorithms, there is an optimization objective based on their properties. For example, the optimization search process of the ant colony algorithm is carried out by searching for food for ants, in contrast to the EO algorithm, which searches for equilibrium states of the search food. However, in the optimization process of the EO algorithm, there is no specific level of concentration to reach the equilibrium state, so the equilibrium state is artificially defined by the four best particles found and the average particle. These five particles help the EO algorithm to perform better in exploration and exploitation, and they all exist in an equilibrium pool, mathematically formulated as follows:
$${\vec{\text{p}}}_{\text{eq},\text{pool}}=\left[{\vec{\text{p}}}_{\text{eq}(1)},{\vec{\text{p}}}_{\text{eq}(2)},{\vec{\text{p}}}_{\text{eq}(3)},{\vec{\text{p}}}_{\text{eq}(4)},{\vec{\text{p}}}_{\text{eq}(\text{avg})}\right]$$ (5)
Step 3: Update method of concentration
  EO algorithms need to find a reasonable balance between development ability and exploration ability, and this process is achieved by balancing turnover $\vec{F}$. In some control volume, the rate of turnover varies with time, assuming $\vec{\lambda }$ is a random vector between 0 and 1.
$$\vec{F}=e^{-\vec{\lambda }\left(t-t_{0}\right)}$$ (6)
  Where *t* is with the increment in iteration, the formula is as follows
$$t={\left(1-\frac{iter}{t_{\text{max}}}\right)}^{\left(a_{2}*\left(\frac{iter}{t_{\text{max}}}\right)\right)}$$ (7)
  Where *iter* and *t*_max_ represent the current iteration number and the maximum iteration number respectively, *a*_*2*_ represents the constant value of the control development capability. In addition, parameter *a*_*1*_ is designed to enhance the diversity and exploration capacity of the population, as follows:
$${\vec{t}}_{0}=\frac{1}{\vec{\lambda }}\text{ln}\left(-a_{1}\text{sign}(\vec{r}-0.5)\left[1-e^{-\vec{\lambda }t}\right)+t\right.$$ (8)
  Generation rate *R* is another parameter used to improve the development operator, and its formula is as follows:
$$\vec{R}={\vec{R}}_{0}*e^{-\vec{\lambda }*\left(t-t_{0}\right)}$$ (9)
$${\vec{R}}_{0}=\vec{\text{RCP}}*(\vec{c_{\text{eq}}}-\vec{\lambda }*\vec{C})$$ (10)
$$\vec{\text{RCP}}=\left\{\begin{array}{ll} 0.5r_{1} & r_{2}>\text{RP}\\ 0 & \text{otherwise} \end{array}\right.$$ (11)
  Where $\vec{\lambda }$ is a random vector between [0,1], *r*_*1*_ and *r*_*2*_ are random numbers between 0,1, and $\vec{\text{RCP}}$ is the control parameter for the generation rate and also has the update process to determine whether the generation rate will be applied to the EO algorithm.
  Finally, the update equation of EO is as follows:
$$\vec{C}=\vec{c_{\text{eq}}}+(\vec{C}-\vec{c_{\text{eq}}})*\vec{F}+\frac{\vec{R}}{\vec{\lambda }*V}*(1-\vec{F})$$ (12)
  Here *V* is assigned 1. For more detailed introduction of EO algorithm, please refer to Faramarzi [43].

### 3.2 Levy Equilibrium Optimizer

**Fig 1:**
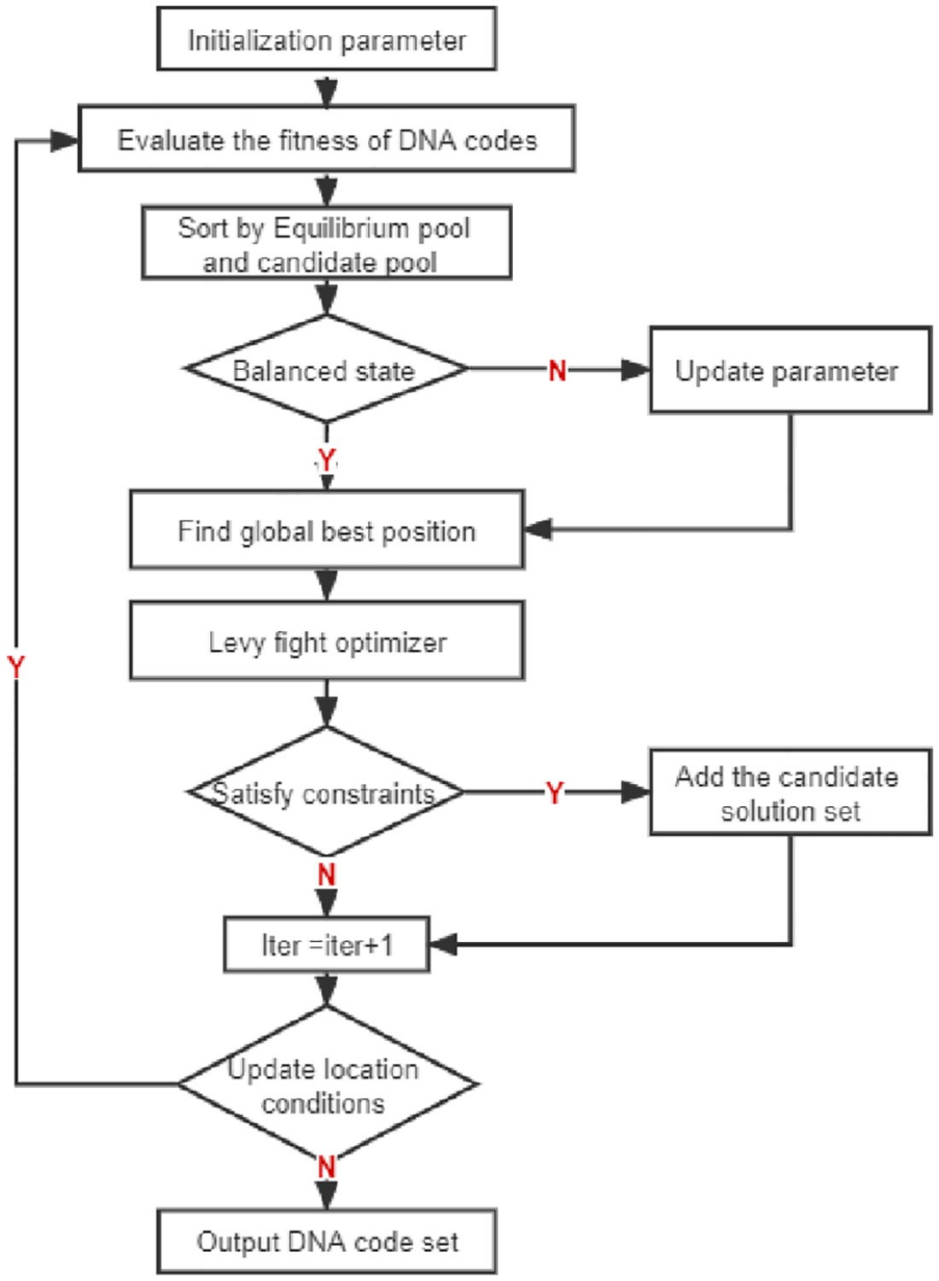
Flow chart of LEO algorithm.

Although the EO algorithm uses parameters such as *a*_*1*_ to enhance the exploration ability of the population, the population richness of the EO algorithm still decreases in later iterations, a situation that is likely to increase the probability of falling into a local optimum, which may be exacerbated in the actual solution process due to more complex conditions. And the individual update mainly depends on the size of the turnover, and then update randomly according to the current optimal global and equilibrium pool. Since the early optimal global value of the algorithm is often too far from the true value, this strategy will increase the probability of the algorithm falling into local optimal, and may lead to a decrease in the convergence speed of the algorithm. A study by Reynolds et al. [44] showed that Drosophila flies explore their environment and search for food during foraging through a series of straight-line flight paths that are often interspersed with abrupt right-angle turns. An intermittent scale-free search model, called Levy, was proposed based on the scale-free flight of Drosophila. And the model was applied to the optimization process and optimal search by the researchers, and it was shown to have good search performance by preliminary results [45]. In LEO algorithm, Levy Flights update strategy is used to replace random update based on current global optimization, which reduces the influence of minimax pool individuals on update mechanism. Therefore, levy flight algorithm was added in the later iteration of the algorithm in this paper to accelerate the convergence of EO algorithm and jump out of local optimum through Levy flight operation. In this paper, levy flight algorithm is used to carry out Levy flight operation on the pool in the late iteration of EO algorithm and process the output of EO algorithm, which can expand the search scope of EO algorithm and obtain a larger code set. By initializing set *S*, determine whether all codes in set *S* and *S*_*EO*_ meet the combination constraint one by one. The flow chart of LEO algorithm is shown in Fig 1.

## 4. Result and analysis

### 4.1 Benchmark function

**Fig 2:**
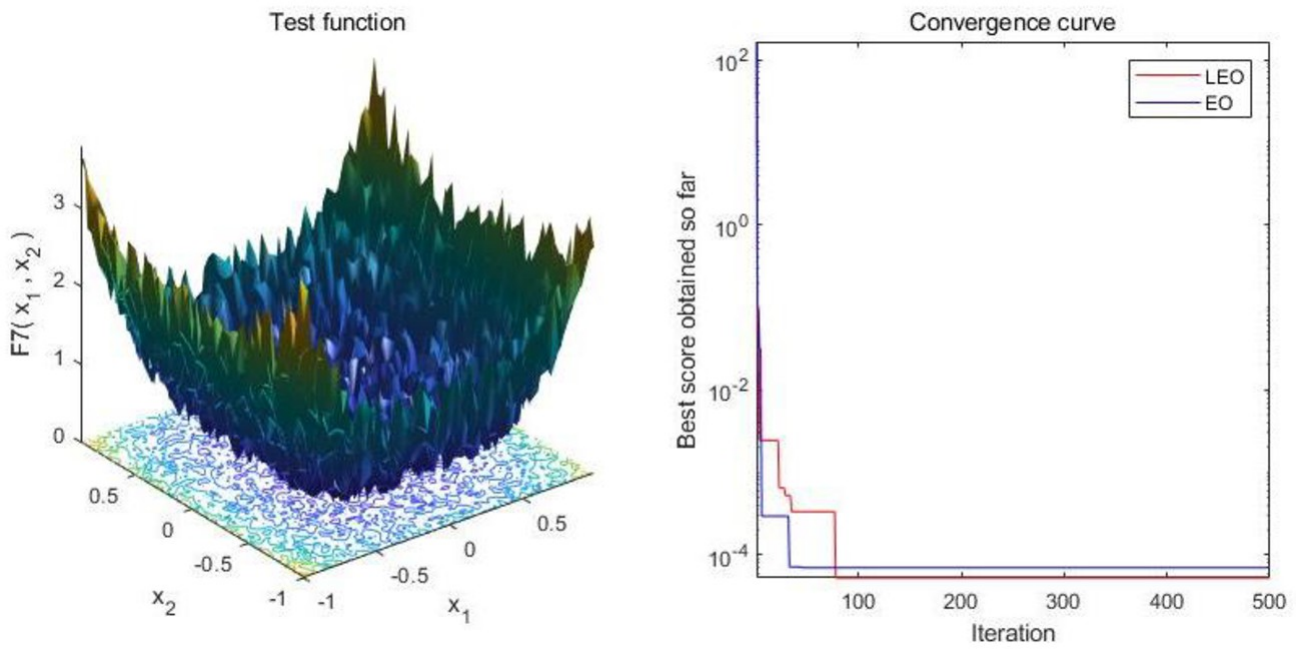
Comparison of convergence curves of LEO and EO algorithms on F7.

In order to verify the performance of the LEO algorithm more clearly, the test function approach is used in this paper. Benchmarking was carried out by using the 13 dominant benchmark functions [46] in Tables 1 and 2. On the one hand, different algorithms target different types of real-world problems, but on the other hand, it is uncertain whether each algorithm achieves the best results for each problem. Since the test functions are simulations of real problems, different algorithms may be suitable for different test functions. Thirteen benchmark functions were chosen, including seven high-dimensional single-peaked functions and six high-dimensional multi-peaked functions. These 13 functions have the ability to reflect most real-world problems, and testing them provides a useful indication of the performance of the algorithm. For the sake of fairness and to improve the reliability of the results and the rigour of the experiments, it is necessary to limit the domain of definition and the number of iterations of the test functions. In order to better illustrate the convergence process of LEO, it can be clearly seen in Fig 2 that in the initial stage, LEO and EO maintain the same iteration efficiency, but in the later stage, LEO converges faster and is closer to the global optimum. This is because Levy flight is LEO jumping out of the local optimum and improving the iteration speed.

**Table 1:** Unimodal benchmark functions.

Function	Dim	Range	F_min_
${\text{F}}_{1}(x)=\sum_{i=1}^{n}X_{i}^{2}$	50	[-100,100]	0
${\text{F}}_{2}(x)=\sum_{i=1}^{n}\left|x_{i}\right|\text{+}\prod_{i=1}^{\text{n}}\left|x_{i}\right|$	50	[-10,10]	0
${\text{F}}_{3}(x)=\sum_{i=1}^{n}{\left(\sum_{j-1}^{i}x_{j}\right)}^{2}$	50	[-100,100]	0
$F_{4}(x)={\text{max}}_{i}\{|x_{i}|,1\leq i\leq n\}$	50	[-100,100]	0
${\text{F}}_{5}(x)=\sum_{i=1}^{n-1}[100{\left(x_{i+1}-x_{i}^{2}\right)}^{2}+{\left(x_{i}-1\right)}^{2}]$	50	[-30,30]	0
${\text{F}}_{6}(x)=\sum_{i=1}^{n}{\left([x_{i}+0.5]\right)}^{2}$	50	[-100,100]	0
$F_{7}(x)=\sum_{i=1}^{n}ix_{i}^{4}+random[0,1)$	50	[-1.28,1.28]	0

**Table 2:** Multi-modal benchmark functions.

Function	Dim	Range	F_min_
$F_{8}(x)=\sum_{i=1}^{n}-x_{i}\;\text{sin}(\sqrt{\left|x_{i}\right|})$	50	[-500,500]	
$F_{9}(x)=\sum_{i=1}^{n}\left[x_{i}^{2}-10\;\text{cos}(2\pi x_{i})+10\right]$	50	[-5.12,5.12]	0
$\begin{array}{l} F_{10}(x)=-20\;\text{exp}(-0.2\sqrt{\begin{array}{cc} \frac{1}{n} & \sum_{i=1}^{n}x_{i}^{2} \end{array}})-\\ \;\text{exp}\left(\begin{array}{cc} \frac{1}{n} & \sum_{i=1}^{n}\text{cos}(2\pi x_{i}) \end{array}\right)+20+e \end{array}$	50	[-32,32]	0
$F_{11}(x)=\frac{1}{4000}\sum_{i=1}^{n}x_{i}^{2}-\prod_{i=1}^{n}x_{i}^{2}\text{cos}\left(\frac{x_{i}}{\sqrt{i}}\right)+1$	50	[-600,600]	0
$\begin{array}{l} F_{12}(x)=\frac{\pi }{n}\left\{10\;\text{sin}(\pi y_{1})+\sum_{i=1}^{n-1}{\left(y_{i}-1\right)}^{2}[1+10{\;\text{sin}}^{2}(\pi y_{i+1})+{\left(y_{n}-1\right)}^{2}]\right\}\\ \;+\sum_{i=1}^{n}u(x_{i},10,100,4)\\ y_{i}=1+\frac{x_{i}+1}{4}\\ u(x_{i},a,k,m)=\left\{\begin{array}{c} k{\left(x_{i}-a\right)}^{m}\begin{array}{cc} & x_{i}>a \end{array}\\ \begin{array}{cc} 0 & -a<x_{i}<a \end{array}\\ k{\left(-x_{i}-a\right)}^{m}\begin{array}{cc} & x_{i}<-a \end{array} \end{array}\right. \end{array}$	50	[-50,50]	0
$F_{13}(x)=0.1\left\{\left.{\text{sin}}^{2}(3\pi x_{1})+\sum_{i=1}^{n}\begin{array}{l} {\left(x_{i}-1\right)}^{2}[1+{\text{sin}}^{2}(3\pi x_{i}+1)]+\\ {\left(x_{n}-1\right)}^{2}[1+{\text{sin}}^{2}(2\pi x_{n})] \end{array}\right\}\right.$	50	[-50,50]	0

After running the 13 test functions for 30 times, the mean and variance of the results were compared with the original algorithm and other representative algorithms. We selected EO, PSO, GWO, GA, GSA and SSA algorithms for comparison, among which EO is the latest work from Mirjalili et al. [29], GA is the earliest and well-performing evolutionary algorithm, PSO is a heuristic algorithm that mimics group behaviors and has group validity, and GSA is a generalization based on physical significance. The maximum number of iterations for these algorithms is set at 500. EO, PSO, GWO, GA, GSA and SSA results are derived from Faramarzi’s work [43]. Tables 3 and 4 list the test functions used.

**Table 3:** Average result of benchmark functions.

F	LEO	EO [43]	PSO [43]	GWO [43]	GA [43]	GSA [43]	SSA [43]
	Ave	Ave	Ave	Ave	Ave	Ave	Ave
F1	0	3.32E-40	9.59E-06	6.59E-28	0.55492	2.53E-16	1.58E-07
F2	0	7.12E-23	0.02560	7.18E-17	0.00566	0.05565	2.66293
F3	0	8.06E-09	82.2687	3.29E-06	846.344	896.534	1709.94
F4	0	5.39E-10	4.26128	5.61E-07	4.55538	7.35487	11.6741
F5	27.93	25.32331	92.4310	26.81258	268.248	67.5430	296.125
F6	0.177	8.29E-06	8.89E-06	0.816579	0.56250	2.5E-16	1.80E-07
F7	5.00E-05	0.001171	0.02724	0.002213	0.04293	0.08944	0.1757
F8	-7216.86	-9016.34	-6075.85	-6123.1	-10546.1	-2821.1	-7455.8
F9	0	0	52.8322	0.31052	30.8229	25.9684	58.3708
F10	8.89E-16	8.34E-14	0.00501	1.06E-13	1.63551	0.06208	2.6796
F11	0	0	0.02381	0.00448	0.56112	27.7015	0.0160
F12	0.0720	7.97E-07	0.02764	0.05343	0.03088	1.79961	6.9915
F13	2.8947	0.029295	0.00732	0.65446	0.36222	8.89908	15.8757

**Table 4:** Standard deviation of benchmark functions.

F	LEO	EO [43]	PSO [43]	GWO [43]	GA [43]	GSA [43]	SSA [43]
	SD	SD	SD	SD	SD	SD	SD
F1	0	6.78E-40	3.35E-05	1.58E-28	1.23010	9.67E-17	1.71E-07
F2	0	6.36E-23	0.04595	7.28E-17	0.01443	0.19404	1.66802
F3	0	1.60E-08	97.2105	1.61E-05	161.499	318.955	11242.3
F4	0	1.38E-09	0.67730	1.04E-06	0.59153	1.74145	4.1792
F5	0.1391	0.169578	74.4794	0.793246	337.693	62.2253	508.863
F6	0.6010	5.02E-06	9.91E-06	0.482126	1.71977	1.74E-16	3.00E-07
F7	4.76E-05	6.54E-04	0.00804	0.001996	0.00594	0.04339	0.0629
F8	734.51	595.1113	754.632	909.865	353.158	493.037	772.811
F9	0	0	16.7068	0.35214	7.57295	7.47006	20.016
F10	0	2.53E-14	0.01257	2.24E-13	0.46224	0.23628	0.8275
F11	0	0	0.02870	0.00665	0.26942	5.04034	0.0112
F12	0.030	7.69E-07	0.05399	0.02073	0.04092	0.95114	4.4175
F13	0.0870	0.035271	0.01050	0.00447	0.30975	7.12624	16.1462

F1-F7 is a high-dimensional single-peak function with global optimality, so it is usually used for general testing of algorithms. F8-F13 has a global optimal and several local optimal, and the number of local optimal solutions increases with the increase of dimension. This increases the difficulty of heuristic algorithm, and can better reflect the optimization speed and jump out of local optimal performance of an algorithm. Tables 3 and 4 show LEO’s performance on the 13 test functions, and for the most part, LEO achieved the best results in the table. However, in the face of complex functions such as F12 and F13, LEO performance is unsatisfactory, which may be that in the face of multi-peak functions, the performance of Levy algorithm is limited, so the optimal solution is not obtained. However, on multi-dimensional unimodal functions, such as F1-5, LEO algorithms find the global optimal solution 0. In order to further illustrate the statistical significance of LEO algorithm, we conducted Wilcoxon test on LEO algorithm, and in most cases, LEO algorithm passed statistical verification. The results are shown in Table 5.

**Table 5:** P values of Wilcoxon rank sum test over 30 runs.

F	LEO	DMVO [40]	KMVO [40]	MVO [40]	GWO [40]	GSA [40]
F1	0.48079	0.52161	0.34817	N/A	0.002827	0.000183
F2	0.54253	0.48536	0.51033	0.009108	0.053903	0.909722
F3	0.5106	0.50454	0.87769	N/A	0.000183	0.000183
F4	0.51411	0.49386	N/A	N/A	0.140465	0.000183
F5	0.53486	0.52008	N/A	0.677585	0.10411	0.005795
F6	0.53806	0.54341	0.58492	N/A	0.000183	0.000182
F7	0.53419	0.47454	0.62052	N/A	0.000183	0.000183
F8	0.51545	0.51805	0.60117	N/A	0.053903	0.000183
F9	0.44637	0.49819	0.26379	0.121225	N/A	0.002827
F10	0.59133	0.47312	0.35904	0.121225	0.001315	N/A
F11	0.43766	0.46246	0.59211	N/A	0.005795	0.000183
F12	0.42515	0.47573	0.48449	N/A	0.025748	0.000183
F13	0.55059	0.4587	N/A	N/A	0.075662	0.000183

### 4.2 lower bound of the DNA storage code set

The DNA coding set with length *n*, hamming distance *d* and meeting hamming distance constraint, GC content constraint and no repeated base constraint is defined as A^*GC*,*NL*^(*n*, *d*, *w*). In Table 5, the results in the table are *4≤n≤ 10*, *3 ≤d≤n* satisfy the lower bound of the constraint. Any algorithm seeking optimization requires a fitness function, so the LEO algorithm uses the sum of the Hamming distances of one of the constraints as a fitness function for the DNA constraint encoding process.


$$fitness=\sum_{i=1}^{n}H(s,S_{i})$$ (13)

As shown in Table 6, we list the results based on the LEO algorithm and compare them with the best results from Li and Limbachiya [47]. The part in bold represents the optimal solution under the same constraints, A represents the best result in Limbachiya and Li, and LEO represents the result in this paper. When n = 9 and d = 4, the size of the DNA storage coding set constructed by the LEO algorithm was 13.2% higher than the results in previous representative work. This is because LEO algorithm uses Generation probability and Equilibrium pool mechanism to balance the process of exploration and development well, and levy flight strategy is used in the late iteration to jump out of local optimum and approach the optimal solution more closely. The results of LEO algorithm provide good initialization, and the balanced pool strategy further extends the results of EO algorithm. More DNA storage codes can reduce the cost consumption of DNA storage system and can perform the same function with the same length. Better quality DNA storage coding can reduce the error rate in the reading and writing process, ensure the overall operation of the DNA storage system, and DNA as a storage medium is also a low-carbon storage method.

**Table 6:** Coding lower bound of A^*GC*,*NL*^(*n*, *d*, *w*).

n\d		3	4	5	6	7	8	9
4	A	12						
	LEO	12						
5	A	20	8					
	LEO	21	8					
6	A	55	21	8				
	LEO	60	21	8				
7	A	125	46	16	6			
	LEO	132	48	16	6			
8	A	364	110	38	15	5	4	
	LEO	373	119	40	16	5	4	
9	A	737	226	71	26	11	5	4
	LEO	743	256	73	30	12	5	4
10	A	1856	546	153	53	22	9	5
	LEO	1900	566	165	57	21	9	5

By comparing the results with those of Limbachiya and Li [47], it is clear that the LEO algorithm yields a significant advantage over the best of them in terms of coding. The LEO algorithm is an intelligent algorithm based on a greedy algorithm that removes the "worst" candidates in each iteration and iteratively removes potential code words to obtain a set of codes that satisfy the requirements. As the algorithm repeats, the altruistic algorithm greedily removes the maximum number of coding words d-1 in the radial range until the distance d of the coding set is minimal. However, altruistic algorithms based on greedy algorithms do not consider the global optimality, but only construct a local optimal solution in a specific sense. Similarly, EORS algorithm also has a random search phase, which is expected to search more valid DNA codes through greedy search strategy, but the time complexity is increased. Therefore, in this work, we use the heuristic algorithm LEO. LEO algorithm is an improvement of EO algorithm based on Levy algorithm and has the advantages of fast convergence speed and high population richness, which can help EO algorithm to converge faster and find the approximate optimal solution.

## 5. Conclusion

This paper proposes a LEO algorithm for DNA storage coding through combinatorial constraints. By approximating the DNA storage coding problem satisfying the constraints to a multi-objective optimization problem, the heuristic algorithm LEO is used to solve the approximate optimal solution of DNA storage coding. Not only can the native advantages of heuristic algorithms for non-linear multi-objective optimizations problems be fully exploited, but the low complexity of constraint encoding is also applied to the field of DNA storage encoding. Encoding that satisfies the constraints reduces the error rate in DNA synthesis and sequencing, as well as the probability of specific hybridization of DNA sequences during PCR. In order to illustrate the superiority of the LEO algorithm proposed in this paper, compared with many convincing algorithms under the benchmark function, the results show that the LEO algorithm has significant advantages in AVE and SD, indicating the effectiveness of the improved algorithm. A larger DNA coding set was constructed under the same combinatorial constraints, and the coding results achieved satisfactory results compared to previous work. The experiments show that in the majority of cases, the coding scheme proposed in this paper achieves satisfactory results compared to the optimal results of Li and Limbachiya, and the lower bound of the coding set is significantly improved, which also illustrates the excellent performance of the LEO algorithm proposed in this paper from the perspective of practical applications. Under the same constraints, the size of the LEO algorithm constructed DNA storage code set is increased by 4–13%. A larger set of stored codes can store more valid information in the same DNA length, reducing costs and improving read and write efficiency. This means that the same performance can be achieved in smaller code lengths, allowing for more efficient and competitive storage of DNA storage systems at a lower cost.

In future work, we will continue to focus on DNA storage coding and continue to study the existing problems of low coding efficiency, low coding quality and insufficient coding set. The intention is to achieve truly fully automated DNA storage as a powerful alternative to traditional silicon-based storage. In addition, the encryption and decryption of image information and text information can be considered for the security of DNA storage, and finally realize the encryption of carbon-based storage and computing integrated equipment similar to silicon-based computer.
